# Vitamin D levels in patients attending a tertiary care hospital in Mogadishu, Somalia: a retrospective review of 28,125 cases

**DOI:** 10.1590/1806-9282.20231100

**Published:** 2024-03-15

**Authors:** Mosab Ahmed Nor, Esra Keles, Mohamed Abdulkadir Hassan-Kadle, Mohamed Abdulahi Hassan, Kursad Nuri Baydili, Hasan Huseyin Eker

**Affiliations:** 1 University of Health Sciences Turkey, Mogadishu Somalia-Turkey Recep Tayyip Erdoğan Training and Research Hospital, Department of Internal Medicine – Mogadishu, Somalia.; 2 University of Health Sciences Turkey, Kartal Lütfi Kırdar City Hospital, Department of Gynecologic Oncology – İstanbul, Turkey.; 3 Abrar University, College of Medicine and Health Science, Center For Digestive and Liver Diseases, SomGastro Clinic – Mogadishu, Somalia.; 4 SIMAD University, Dr. Sumait Hospital, Faculty of Medicine and Health Sciences, Department of Internal Medicine – Mogadishu, Somalia.; 5 University of Health Sciences Turkey, Hamidiye Faculty of Medicine, Department of Biostatistics – İstanbul, Turkey.; 6 University of Health Sciences Turkey, Hamidiye Faculty of Medicine, Department of Public Health – İstanbul, Turkey.; 7 University of Health Sciences Turkey, Mogadishu Somalia-Turkey Recep Tayyip Erdoğan Training and Research Hospital, Department of Public Health – Mogadishu, Somalia.

**Keywords:** Vitamin D, Vitamin D deficiency, Somalia

## Abstract

**OBJECTIVE:**

The objective of this study was to identify the prevalence and risk factors for vitamin D deficiency among patients attending a tertiary hospital in Mogadishu, Somalia.

**METHODS:**

This retrospective study examined the results of serum 25-hydroxy-vitamin D tests of 28,125 patients admitted to Somalia Mogadishu-Turkey Training and Research Hospital between January 2017 and December 2021. Vitamin D insufficiency is defined as 20–30 ng/mL, deficiency as 10–19 ng/mL, and severe deficiency as <10 ng/mL.

**RESULTS:**

A total of 28,125 patients with a mean age of 44.27±20.4 years were included in the study. The majority of patients were in the age group of 19–40 years. The mean serum level of 25-hydroxy-vitamin D was 28.42±15.34 ng/mL. Of the patients included in the study, 5.8% (1,618/28,125) had vitamin D sufficiency, 6.5% (1,826/28,125) had vitamin D insufficiency, 41.8% (11,761/28,125) had vitamin D deficiency, and 45.9% (12,920/28,125) had severe vitamin D deficiency. The mean serum 25-hydroxy-vitamin D levels were lower in females than in males (p<0.001).

**CONCLUSION:**

The study indicated a high prevalence of vitamin deficiency among patients attending the largest tertiary care hospital, particularly female patients and older people. It is recommended to develop educational and awareness programs, and campaigns to reduce vitamin D deficiency in the population, especially those at high risk.

## INTRODUCTION

Vitamin D deficiency is one of the major public health problems affecting over 1 billion people worldwide^1^. Vitamin D is essential for every individual to lead a healthy life. Vitamin D is a prohormone and fat-soluble vitamin. Even though a small amount of vitamin D is obtained from food, the major amount of vitamin D is obtained from exposure to the ultraviolet-B (UVB) component of sunlight^2^.

There is an ongoing debate over the determination of serum 25-hydroxy-vitamin D (25(O.H.)D) concentration for defining the vitamin D status^1,3^. Additionally, the vitamin D range varies between populations and depends upon many factors. Therefore, the Scientific Advisory Committee on Nutrition (SACN) has recently contributed an excellent overview of the current vitamin D status as well as defined recommendations for adequate 25(O.H.) D concentrations in the general population^4^.

Circulating 25(O.H.) D is a reliable indicator of vitamin D nutritional status. Recently, many studies have used 30 ng/mL as a cutoff value, and most experts now recommend ≥ff ng/mL as the normal level of 25(O.H.) D, 20–29 ng/mL as vitamin D insufficiency, and ecomng/mL as vitamin D deficiency^5^.

Many studies have shown that inadequate levels of vitamin D can lead to a variety of negative health conditions, from rickets and osteoporosis to obesity, type 2 diabetes, hypertension, depression, fibromyalgia, and Parkinson's disease^6,7^. Vitamin D deficiency may even contribute to the development of cancers as well as cardiovascular disease, Alzheimer's disease, stroke, autoimmune diseases, pelvic floor diseases, and periodontal disease^7^.

Many studies have been conducted on vitamin D in different age groups, diseases, ethnic groups, and populations. However, our knowledge on vitamin D levels among people in Somalia is limited. To be the baseline for future research, to the best of our knowledge, this is the first comprehensive study to be reported from Somalia. Therefore, we aimed to examine the prevalence and risk factors of vitamin D deficiency among patients attending a tertiary hospital in Somalia.

## METHODS

This retrospective study was carried out by examining the results of serum 25(O.H.) D tests of 28,125 patients who were admitted to Somalia Mogadishu-Turkey Training and Research Hospital between January 2017 and December 2021. Our institution is the largest hospital in Somalia and provides a tertiary level of care to more than 2 million people in Mogadishu. The city of Mogadishu, where the study was conducted, is located at 2°4’ north, 45°22’ east latitude, with an average daily and annual sunlight of 8.4 and 3066 h, respectively, and receives 70% of possible sunlight.

The data were retrieved from the hospital's electronic database following the approval of the Research Ethics Committee of Somalia Mogadishu-Turkey Recep Tayyip Erdogan Training and Research Hospital (Approval number: 21.09.2021, MSTH/7426). Informed consent from study participants was waived due to the retrospective nature of the study. Abstracted data included age, gender, and year. Repeated measurements obtained from the same patient in different admissions were excluded from the analysis.

The 25(O.H.) D levels were measured by the chemiluminescent immunoassay method with the Mindray CL-2000i Chemiluminescence Immunoassay System in the biochemistry laboratory. According to the Endocrine Society Clinical Practice Guidelines, patients were divided into four categories based on their serum 25(O.H.) D levels: vitamin D sufficiency was defined as >30 ng/mL, insufficiency as 20–30 ng/mL, deficiency as 10–19 ng/mL, and severe deficiency as <10 ng/mL^8,9^.

### Statistical analysis

Analysis was carried out using Statistical Package for the Social Science (IBM SPSS, Version 25.0. Armonk, NY: IBM Corp.) for the Windows software. Data were expressed as frequency (n) and percentage (%) for qualitative variables and arithmetic mean and standard deviation values (Mean±SD) for quantitative variables. The χ² test or Fisher's exact test was used for categorical variables, and the independent-sample t-test was used for continuous variables. The one-way analysis of variance (ANOVA) test followed by the Tukey post-hoc test was performed for multiple comparisons. The type I error rate was set at 0.05. A p<0.05 was considered statistically significant.

## RESULTS

A total of 28,125 patients were enrolled in the study. The mean age of patients was 44.27±20.4 years, and 59.8% were females. The majority of patients were in the age group of 19–40 years. The mean serum level of 25(O.H.) D was 28.42±15.34 ng/mL. Of the patients included in the study, 5.8% (1,618/28,125) had vitamin D sufficiency, 6.5% (1,826/28,125) had vitamin D insufficiency, 41.8% (11,761/28,125) had vitamin D deficiency, and 45.9% (12,920/28,125) had severe vitamin D deficiency. The baseline characteristics of the patients are shown in Table 1.

**Table 1:** Baseline characteristics of the patients (n=28125).

		n (%)
Year		
	2017	5043 (17.9)
	2018	5285 (18.8)
	2019	5153 (18.3)
	2020	5615 (20)
	2021	7029 (25)
Gender		
	Male	11301 (40.2)
	Female	16824 (59.8)
Age groups (years)		
	0–12	1491 (5.3)
	13–18	1083 (3.9)
	19–40	9332 (33.2)
	41–60	8530 (30.3)
	>60	7689 (27.3)
	Age (Mean ± SD)	44.27±20.4
25(O.H.) D (ng/mL)		
	<10	12920 (45.9)
	10–19	11761 (41.8)
	20–30	1826 (6.5)
	>30	1618 (5.8)
	25(O.H.) D level (Mean ± SD)	28.42±15.34

25(O.H.) D: 25-hydroxy-vitamin D.

Table 2 presents the differences in mean 25(O.H.) D levels between groups stratified by age, gender, and year. When the mean serum 25(O.H.) D levels were evaluated according to the years, it was highest in 2017 and 2021 and was lowest in 2018 (p<0.001). The mean serum 25(O.H.) D levels were 33.18±16.74 ng/mL for the males and 25.23±13.4 ng/mL for the females. The mean serum 25(O.H.) D levels were lower in females than in males (p<0.001). The mean serum 25(O.H.) D levels according to age groups were as follows: 37.67 ng/mL for 0–12 years of age, 25.86 ng/mL for 13–18 years of age, 26.17 ng/mL for 19–40 years of age, 27.78 ng/mL for 41–60 years of age, and 30.44 ng/mL for >60 years of age. The mean levels of 25(O.H.) D among children 0–12 years of age were significantly higher than those found for the other age groups (p<0.001).

**Table 2:** Patient characteristics stratified by mean serum 25-hydroxyvitamin D level.

Characteristics		Mean serum 25-hydroxyvitamin D level	p-value
Year			
	2017	30.8±14.98	<0.001*
	2018	24.52±12.86	
	2019	26.21±13.97	
	2020	28.75±14.87	
	2021	31.01±17.61	
Gender			
	Male	33.18±16.74	<0.001*
	Female	25.23±13.4	
Age (years)			
	0–12	37.67±18.85	<0.001*
	13–18	25.86±14.49	
	19–40	26.17±13.48	
	41–60	27.78±14.59	
	>60	30.44±16.64	

* p<0.05.

The prevalence of vitamin D severe deficiency (<10 ng/mL) was lowest in 0–12 years of age (23.8%), while the highest rates of prevalence were noted in females (54.8%), in those 19–40 years of age (51.3%), and 2018 (57.1%). A comparison of sociodemographic characteristics according to the serum 25(O.H.) D level is shown in Table 3.

**Table 3:** Comparison of sociodemographic characteristics of the study population according to the serum 25-hydroxyvitamin D level.

25(OH) D level intervals (ng/mL)		<10 ng/mL	10–19 ng/mL	20–30 ng/mL	>30 ng/mL	p-value
Year						
	2017	1965 (39)	2580 (51.2)	477 (9.5)	21 (0.4)	<0.001*
	2018	3019 (57.1)	2098 (39.7)	141 (2.7)	27 (0.5)	
	2019	2792 (54.2)	2108 (40.9)	211 (4.1)	42 (0.8)	
	2020	2289 (40.8)	2320 (41.3)	343 (6.1)	663 (11.8)	
	2021	2855 (40.6)	2655 (37.8)	654 (9.3)	865 (12.3)	
Gender						
	Male	3698 (32.7)	5689 (50.3)	1208 (10.7)	706 (6.2)	<0.001*
	Female	9222 (54.8)	6072 (36.1)	618 (3.7)	912 (5.4)	
Age groups (years)						
	0–12	355 (23.8)	779 (52.2)	241 (16.2)	116 (7.8)	<0.001*
	13–18	580 (53.6)	384 (35.5)	54 (5)	65 (6)	
	19–40	4788 (51.3)	3628 (38.9)	389 (4.2)	527 (5.6)	
	41–60	4012 (47)	3597 (42.2)	491 (5.8)	430 (5)	
	>60	3185 (41.4)	3373 (43.9)	651 (8.5)	480 (6.2)	

* p<0.05.

## DISCUSSION

Vitamin D deficiency, which is becoming a serious health problem worldwide, is an issue that affects many individuals of all ages and genders. The overwhelming frequency of vitamin D deficiency among Somali patients at this largest tertiary hospital in Mogadishu, Somalia, is the major finding of this 5-year retrospective study. As a result, our study found that the prevalence of vitamin status levels varied depending on their thresholds; the highest prevalence was severe vitamin D deficiency, which accounted for 45.9% (12,920/28,125), followed by vitamin D deficiency, which accounted for 41.8% (11,761/28,125). The other vitamin D thresholds had a low prevalence of 5.8% (1,618/28,125) in vitamin D sufficiency and 6.5% (1,826/28,125) in vitamin D insufficiency. In our study, the mean serum level of 25(O.H.) D was 28.42±15.34 ng/mL. A study conducted in Riyadh, Saudi Arabia, showed a mean serum 25(O.H.) D level of 35.5±30.6 ng/mL^10^. Another study in Kathmandu, Nepal, found that the total mean serum vitamin D was 19.69±13.68 ng/mL^11^. The findings of this study showed that vitamin D deficiency among Somali patients attending this largest tertiary care hospital is alarmingly high.

Although Somalia is in one of the sunniest parts of the world, our study has similar vitamin D status problems to some parts of Africa, Asia, and the Middle East^12^. A recent systematic review including 195 studies from 44 countries worldwide which used the same cutoff points in our study presented that 88.1% had a mean of 25(O.H.) D values below 30 ng/mL^13^, whereas another recent systematic review and meta-analysis study found that one in five people living in Africa had a low 25(O.H.) D concentration using a less than 30 nmol/L cutoff point^3^. In Saudi Arabia, vitamin D deficiency and insufficiency reached 67.8%^10^. In Benghazi, Libya, the estimated vitamin D deficiency was 76.1.1%, and insufficiency was 15.2%^14^. In Kathmandu, Nepal, vitamin D deficiency was 69.6%, and insufficiency was 16%^11^. In Lebanon, vitamin D deficiency was 63%, and insufficiency was 20.5%^15^. In Egypt, the estimated prevalence was 77% for vitamin D deficiency and 15% for vitamin D insufficiency^16^.

According to the gender variation of vitamin D deficiency in this study, the female population (59.8%) was more predominant than the male population (40.2%). Globally, the female gender is one of the most important predictors of vitamin D deficiency^13,14^. The increased frequency seen in females was comparable with other studies in Saudi Arabia (78.1%)^10^, Libya (58.8%)^14^, Nepal (76.1%)^11^, and Bangladesh (46%)^17^. This finding was due to some factors that females predominate for vitamin D deficiency. Females are 2.8 times more likely to develop vitamin D deficiency than males. Other factors, such as cultural factors (clothing styles), reduced outdoor activities, aggressive sun protection, and low vitamin D intake, could contribute to vitamin D deficiency^9,14^.

This study found that the mean age was 44.27±20.4 years. The majority of patients were in the age group of 19–40 years. The mean age was 36.2±0.9 years in Benghazi, Libya^14^, 40.5±14.4 years in Kathmandu, Nepal^11^, 47±16.3 years in Chattogram, Bangladesh^18^, and 46.9±16.3 in Riyadh, Saudi Arabia^10^.

Our study showed that the mean serum vitamin D concentration among genders was 33.18±16.74 ng/mL in males and 25.23±13.4 ng/mL in females. The mean level of serum vitamin D concentrations was lower in females than in males. In Benghazi, Libya, a study showed that the mean serum vitamin D concentrations by gender were 15.4 ng/mL (95%CI 14.6–16.2) in males and 13.2 ng/mL (95%CI 12.5–13.9) in females^14^. A study similar to our study conducted in Kathmandu, Nepal, showed that the mean serum vitamin D concentration by gender was 22.38±17.07 ng/mL in males and 18.89±15.25 ng/mL in females^11^.

Vitamin D deficiency was seen in 54.8% of female patients and 51.3% in the group aged 19–40 years, while the least of the patients with vitamin D levels were found in the group aged 13–18 years. Various studies reported a similar observation of lowering vitamin D status levels among females and older people, such as studies in Saudi Arabia, Libya, and Nepal^10,11,14^.

The observation of various reports of older people or increased ages that have lowered vitamin D serum concentration is because the older group might not be having vitamin D and calcium supplements or decreased dietary intake, diminished sunlight exposure, reduced skin thickness, impaired intestinal absorption, and impaired hydroxylation of vitamin D in liver and kidney^15-17^. Another necessary explanation is that those younger ages in this study have higher vitamin D levels than older ages where the MENA region (the Middle East/Africa) generally spends more time outdoors compared with other age groups with lower vitamin D levels^13,14,18^. Vitamin D deficiency is predominant in females due to a diet lack of calcium and vitamin D intake, lack of exposure to sunlight due to indoor lifestyle of females, low education levels, and the whole body covering among Muslim women^19,20^.

### Limitations

Several limitations of the study need to be acknowledged. First, the study findings were limited by the use of a retrospective design. Thus, it did not represent the whole population. Second, the study could not analyze the related risk factors and variables that are important in vitamin D status. Notwithstanding these limitations, this study represents the first comprehensive assessment of vitamin D levels in Somali people living in Somalia. Additionally, the large sample size and inclusion of all age groups removed selection bias and increased the generalizability of the results.

## CONCLUSION

The study highlighted a high prevalence of vitamin D deficiency among patients attending our largest tertiary care hospital, particularly among female and older people. To reduce vitamin D deficiency, we recommend developing intensive educational and awareness programs and campaigns to increase the population knowledge and limit the spread of vitamin D deficiency that engulfs the nation. Furthermore, a large-scale, multicentric, or community-based study should be conducted in the near future to determine the more accurate prevalence and to assess the factors that contribute to the vitamin D level of different modifiable and non-modifiable factors to the health problem of the hypovitaminosis-D burden.

## Data Availability

The dataset used and analyzed in the study is available from the corresponding author upon reasonable request.
